# Deviant spontaneous neural activity as a potential early-response predictor for therapeutic interventions in patients with schizophrenia

**DOI:** 10.3389/fnins.2023.1243168

**Published:** 2023-08-31

**Authors:** Huan Jing, Chunguo Zhang, Haohao Yan, Xiaoling Li, Jiaquan Liang, Wenting Liang, Yangpan Ou, Weibin Wu, Huagui Guo, Wen Deng, Guojun Xie, Wenbin Guo

**Affiliations:** ^1^Department of Psychiatry, The Third People's Hospital of Foshan, Foshan, Guangdong, China; ^2^Department of Psychiatry, National Clinical Research Center for Mental Disorders, and National Center for Mental Disorders, The Second Xiangya Hospital of Central South University, Changsha, Hunan, China

**Keywords:** schizophrenia, regional homogeneity, support vector machine, support vector regression, magnetic resonance imaging, brain

## Abstract

**Objective:**

Previous studies have established significant differences in the neuroimaging characteristics between healthy controls (HCs) and patients with schizophrenia (SCZ). However, the relationship between homotopic connectivity and clinical features in patients with SCZ is not yet fully understood. Furthermore, there are currently no established neuroimaging biomarkers available for the diagnosis of SCZ or for predicting early treatment response. The aim of this study is to investigate the association between regional homogeneity and specific clinical features in SCZ patients.

**Methods:**

We conducted a longitudinal investigation involving 56 patients with SCZ and 51 HCs. The SCZ patients underwent a 3-month antipsychotic treatment. Resting-state functional magnetic resonance imaging (fMRI), regional homogeneity (ReHo), support vector machine (SVM), and support vector regression (SVR) were used for data acquisition and analysis.

**Results:**

In comparison to HCs, individuals with SCZ demonstrated reduced ReHo values in the right postcentral/precentral gyrus, left postcentral/inferior parietal gyrus, left middle/inferior occipital gyrus, and right middle temporal/inferior occipital gyrus, and increased ReHo values in the right putamen. It is noteworthy that there was decreased ReHo values in the right inferior parietal gyrus after treatment compared to baseline data.

**Conclusion:**

The observed decrease in ReHo values in the sensorimotor network and increase in ReHo values in the right putamen may represent distinctive neurobiological characteristics of patients with SCZ, as well as a potential neuroimaging biomarker for distinguishing between patients with SCZ and HCs. Furthermore, ReHo values in the sensorimotor network and right putamen may serve as predictive indicators for early treatment response in patients with SCZ.

## 1. Introduction

Schizophrenia (SCZ) is a severe mental disorder that is among the top causes of disability worldwide. It affects approximately 1% of the global population and is characterized by psychotic symptoms such as hallucinations, delusions, and speech disorders. Hallucinations can take various forms, including auditory, visual, and tactile. Among individuals with SCZ, auditory hallucinations are the most frequently reported type (Marder and Cannon, 2019; Jauhar et al., 2022; Xu et al., 2022). Men have a higher risk of developing SCZ compared to women, with an estimated lifetime prevalence rate ranging from 0.3 to 0.7% (Hyman et al., 2005; Moreno-Kustner et al., 2018; Onitsuka et al., 2022). The prevalence of SCZ has been observed to peak at around age 20.5, and among individuals aged 25 and older, the prevalence is 47.4% (Solmi et al., 2022). Researches have indicated that individuals diagnosed with SCZ have a significantly lower life expectancy compared to the general population, with a difference of approximately 15–20 years. Furthermore, this gap in mortality rates is continuing to widen (Tanskanen et al., 2018). Environmental factors, such as social isolation, childhood trauma, urban living, and ethnicity, are considered to be among the multifactorial causes of SCZ (Diaz-Castro et al., 2021; Srivastava et al., 2021). SCZ may have its origins *in utero*, with obstetric potential risk factors including complications such as low birth weight, gestational diabetes, bleeding, asphyxia, and emergency caesarean section. Additionally, fetal disorders such as stress and infection during the second trimester have also been identified as risk factors (Thomas et al., 2001; Buoli et al., 2016). SCZ is widely recognized as a highly heritable disorder, with a estimate reaching as high as 80%. Abnormal brain networks and neural developmental defects are believed to contribute to the inability to integrate neural processes, a hallmark of SCZ (Legge et al., 2021; Zhu et al., 2021). SCZ has caused significant suffering for individuals with the disorder as well as their relatives. However, due to lack of objective indicators, accurately diagnosing and effectively treating SCZ remain major challenges.

Receiving an accurate diagnosis and appropriate treatment is of utmost importance for individuals with SCZ (Xie et al., 2020; Galinska-Skok and Waszkiewicz, 2022). Previous research has suggested a potential link between immune biomarkers and the progression of SCZ, as well as the effectiveness of treatment (Kuloglu et al., 2016; Kozlowska et al., 2021; Li et al., 2022; Ma et al., 2022). In the future, diagnostic and prognostic biomarkers may be utilized to enhance the accuracy of diagnosis, monitoring of therapy, and prediction of treatment outcomes in individuals with SCZ (Perkovic et al., 2017). Neuroimaging holds great promise as a tool for developing biomarkers of SCZ, as it enables the capture of phenotypic variations in molecular and cellular disease targets, as well as brain circuits (Acar et al., 2019; Kraguljac et al., 2021). Numerous neuroimaging studies have aimed to elucidate the neurobiological underpinnings of SCZ and differentiate neuroimaging characteristics between individuals with SCZ and healthy controls (HCs), as well as predict early treatment response. Studies have shown that individuals with SCZ exhibit increased neural activity in the frontal cortex and decreased resting-state functional connectivity (FC) patterns between the left middle frontal gyrus (MFG) and the left medial superior frontal gyrus (MFSG), which have been associated with cognitive impairment (Yu et al., 2022). After treatment, individuals with SCZ have been observed to exhibit an increase in FC between hippocampus and brain networks associated with cognitive function (Jiang et al., 2019). Following treatment with the antipsychotic medication olanzapine, individuals with SCZ have been observed to exhibit increased FC in the default-mode network (DMN) and sensorimotor circuits of the brain. Conversely, reduced FC has been noted in the left superior temporal gyrus (STG) (Guo et al., 2017). Wang et al. (2017) found that individuals with SCZ exhibited increased FC in the right superior temporal gyrus, right medial frontal gyrus, and left superior frontal gyrus, and decreased FC in the right posterior cingulate/anterior cuneus, right cerebellar anterior lobe, and left insular lobe following treatment. Several articles have reviewed neuroimaging studies investigating changes in neural function and structure in individuals with SCZ following treatment. These studies consistently demonstrate that treatment often impacts the brains of individuals with SCZ (Kani et al., 2017; Dabiri et al., 2022). Although treatment-induced early neuroimaging changes have been observed in individuals with SCZ, it is not yet clear whether these changes are directly linked to improvements in the patient’s clinical status. Resting-state functional magnetic resonance imaging (rs-fMRI) has become a valuable tool for investigating brain functional connectivity and is a non-invasive neuroimaging technology (Palacios et al., 2013). FC patterns are identified by coherent oscillations in the resting-state imaging data, which typically range from 0.01 to 0.1 Hz (Yan et al., 2022a).

Regional homogeneity (ReHo) is a widely employed metric in rs-fMRI that quantifies the similarity of time series between a specific voxel and its neighboring voxels (Zang et al., 2004). The ReHo method is a reliable measure of brain activity that can be used to investigate changes in brain function due to normal development and pathological conditions. Additionally, the ReHo method has shown a high test–retest reliability, making it a useful tool for longitudinal studies (Zuo et al., 2013). Previous studies using the ReHo method have shown extensive anomalies in ReHo in individuals with SCZ. For example, Huang et al. (2022) found that compared to HCs, individuals with SCZ exhibited increased ReHo values in the right frontal gyrus and decreased ReHo values in the right anterior cingulate cortex (ACC), left middle occipital gyrus (MOG), left precuneus, right posterior cingulate cortex (PCC), and right superior occipital gyrus. In addition, in patients with SCZ, ReHo in the right superior frontal gyrus (Chen et al., 2013; Xiao et al., 2017), right superior temporal gyrus (Xiao et al., 2017), right middle frontal gyrus (MFG), superior frontal gyrus (SFG) (Yan et al., 2020), right anterior cingulate gyrus and left medial superior frontal gyrus (Gao et al., 2015) increased, and ReHo in the left fusiform gyrus, left superior temporal gyrus, left posterior central gyrus, right anterior central gyrus (Xiao et al., 2017), and left superior occipital gyrus decreased. However, it remains unknown whether and how the abovementioned abnormalities change with antipsychotic treatment in SCZ.

Support vector machine (SVM) is a type of supervised machine learning that applies multivariable pattern recognition technology to predict various conditions or outcomes, such as psychosis, based on neuroanatomical markers (Shan et al., 2021). Support vector regression (SVR) is a type of supervised machine learning that is particularly effective in handling nonlinear regression tasks. It achieves this by projecting original features into kernel space, where the data may be linearly separated (Ben-Hur et al., 2008). We employed SVM to investigate the presence of baseline aberrant ReHo values in individuals with SCZ as a potential neuroimaging biomarker for diagnosis. Additionally, SVR was utilized to investigate the potential of baseline aberrant ReHo values as neuroimaging biomarkers for early treatment response in individuals with SCZ.

Our hypothesis is that individuals with SCZ will display aberrant baseline ReHo values, which will be altered after 3 months of drug treatment. Furthermore, we postulate that these abnormal baseline ReHo values have the potential to function as neuroimaging biomarkers for both diagnosing SCZ and predicting early treatment response in individuals with the disorder.

## 2. Materials and methods

### 2.1. Participants

This study included a total of 107 participants, comprising 56 individuals with SCZ and 51 HCs. HCs were recruited from physical examination centers and local communities, while individuals with SCZ were recruited from the Department of Psychiatry at the Third People’s Hospital of Foshan.

The inclusion criteria for individuals with SCZ were as follows: (1) Meeting the diagnostic criteria for SCZ as outlined in the Diagnostic and Statistical Manual of Mental Disorders-5 (DSM-5); (2) Aged between 18 and 55 years old; (3) Right-handed; (4) Education level of at least 6 years.

The inclusion criteria for HCs were as follows: (1) No personal or family history of mental disorders; (2) Aged between 18 and 55 years old; (3) Right-handed; (4) Education level of at least 6 years.

Exclusion criteria were as follows: (1) Obvious brain lesions, including cerebral infarction, hemorrhage, or intracranial mass; (2) A history of other mental disorders, such as anxiety, depression, bipolar disorder, mental retardation, and eating disorders; (3) Alcohol or substance addiction; (4) Any contraindication for MRI, such as metal implants or claustrophobia; (5) Metabolism-related diseases, such as hypertension, diabetes, hypothyroidism, or hyperthyroidism; (6) Pregnancy or lactation in women.

The psychological status and cognitive status of the subjects were measured by the Positive and Negative Symptom Scale (PANSS), Hamilton Depression Scale (HAMD), Hamilton Anxiety Scale (HAMA), Insight and Treatment Attitudes Questionnaire (ITAQ), Social Disability Screening Schedule (SDSS), Wisconsin Card Sorting Test (WCST), Repeatable Battery for the Assessment of Neuropsyehological Status (RBANS), Stroop Color-Word Test (SCWT), and Simplified Coping Style Questionnaire (SCSQ).

This study has obtained approval from the Medical Ethics Committee of Foshan Third People’s Hospital (Foshan Mental Health Center). In addition, informed consent was obtained from all participants or their legal guardians.

### 2.2. Procedure

At baseline, all participants underwent a 3.0 T brain MRI scan and provided clinical information, including Body Mass Index (BMI), Thyroid Stimulating Hormone (TSH3UL), Free Triiodothyronine (FT3), Free Thyroxine (FT4), triglycerides (TG), Cholesterol (CHOL), High-Density Lipoprotein (HDL), Low-Density Lipoprotein (LDL), Fasting Blood Glucose (FBG), and Heart Rate (HR).

Following the baseline assessment, individuals with SCZ underwent a 3-month antipsychotic treatment period and subsequently underwent a follow-up MRI scan. Their clinical symptoms were evaluated using the PANSS, HAMD, HAMA, and ITAQ at both baseline and the endpoint.

The treatment approaches for SCZ primarily involve a combination of medication and physical therapies. Medications commonly used include Olanzapine, Risperidone, Lorazepam, Aripiprazole, and others. Physical therapies encompass transcranial magnetic stimulation (TMS) for brain functional modulation and neurofeedback therapy utilizing electroencephalography (EEG).

### 2.3. Measures

The PANSS is a 30-item scale that includes a Positive Scale (P) with 7 items, a Negative Scale (N) with 7 items, and a General Psychopathology Scale (G) with 16 items. Each item is rated on a 7-point scale ranging from 1 to 7, with the sum of the scores for each subscale used to obtain the total score. Higher scores on the scale indicate more severe symptoms of SCZ (Kay et al., 1987). The HAMD is a widely used tool for evaluating the severity and effectiveness of treatment for depression. Scores are obtained before and after treatment based on conversation and observation, with higher scores indicating more severe symptoms. Similarly, the HAMA is used to assess anxiety symptoms, with each of the 14 items scored between 0 and 4. Higher scores indicate more severe anxiety symptoms (Bagby et al., 2004). The ITAQ is an 11-item questionnaire used to evaluate patients’ understanding of their mental illnesses and treatment requirements. The overall score, measured on a 3-point Likert scale, reflects the patient’s understanding of the illness and its treatments, with higher scores indicating better understanding (Kemp and Lambert, 1995). SDSS is a 10-item screening schedule that assigns 0–2 points to each item. The total score is the sum of all items, with higher scores indicating more severe social function defects in the patient (Yan et al., 2022b). WCST is a cognitive task that evaluates executive function using 64 cards that vary in shape, color, and number (Nieuwenstein et al., 2001). RBANS assesses cognitive function across five dimensions: immediate memory, language, attention, delayed memory, and visual spatial construction. The higher the score, the better the cognitive function (Olaithe et al., 2019). SCWT is a neuropsychological test that assesses an individual’s ability to manage conflicting stimulus attributes when presented with specific stimuli. SCSQ is a tool used to evaluate an individual’s coping ability. It includes 20 questions across two dimensions of positive and negative coping (Fan et al., 2022).

### 2.4. Imaging data acquisition and preprocessing

Resting-state fMRI data were acquired using a GE 3.0 T scanner (GE 3.0 T Signa Pioneer) with the following parameters: repetition time/echo time = 2000/30 ms, 36 slices, 64 × 64 matrix, flip angle 90°, field of view 24 cm, slice thickness 4 mm, no gap, 250 volumes (500 s). Subjects were instructed to remain still, close their eyes, and stay awake while wearing soft earplugs and foam pads to reduce scanner noise and head movements. With the Data Processing Assistant for Resting-State fMRI (DPARSF) software in MATLAB, preprocessing included slice timing correction, head motion correction, normalization to 3 × 3 × 3 mm^3^, and maximum displacement and angular motion limits of 2 mm and 2°, respectively (Chao-Gan and Yu-Feng, 2010). Linear trend removal and band-pass filtering with a frequency range of 0.01–0.08 Hz were applied (Song et al., 2011).

### 2.5. ReHo calculation

The ReHo brain map was generated by first calculating the Kendall coefficient of time series consistency between each voxel and its 26 neighboring voxels. The ReHo value of each voxel was then normalized by subtracting the average ReHo value of the whole brain and dividing it by the standard deviation. This was followed by calculating the KCC-ReHo values in all individual voxel directions, which were then normalized to KCC-ReHo z-values for further analysis (Zuo et al., 2013). A Gaussian kernel with a full width at half maximum of 4 mm was employed to smooth the data. This step aimed to reduce the influence of deformation and noise during the normalization process, enhance the signal-to-noise ratio and statistical efficiency, and improve the image quality.

### 2.6. Statistical analysis

Demographic data differences between patients with SCZ and HCs were analyzed using two-sample t-tests and chi-square tests as appropriate with SPSS 25.0 software. Paired t-tests were used to compare clinical symptoms of patients with SCZ at baseline and endpoint, with statistical significance set at *p* < 0.05 (two-tailed).

To analyze the imaging data, the DPARSF software package was used. Two-sample *t*-tests were performed on individual normalized ReHo maps to identify clusters with abnormal ReHo values in patients with SCZ at baseline compared to HCs. Covariates including mean framewise displacement, gender, age, and years of education, were used in the analysis. The ReHo values before and after treatment were compared using paired *t*-tests with the mean framewise displacement as covariates. Multiple comparisons were corrected using the Gaussian random field (GRF) theory with a cluster significance of *p* < 0.05 (two-tailed) and voxel significance of *p* < 0.001 (two-tailed).

Pearson/Spearman correlation analyses were conducted to evaluate the relationship between abnormal ReHo values and clinical data of patients with SCZ at baseline.

### 2.7. SVM analysis

The study employed SVM analysis by utilizing the LIBSVM software^1^ in MATLAB, to determine the capability of abnormal ReHo values extracted from specific brain regions to differentiate between HCs and patients with SCZ. The “leave one-out” method was utilized in the analysis. The SVM algorithm utilizes the FC values in the training set to discern dissimilarities between groups and identifies a maximum-margin hyperplane that separates the two groups. Once training is finished and the decision function is determined, it predicts the class label of each sample in the test set. These steps are iterated until each sample has been used as a testing sample. During each cross-validation, we calculate three performance measures (accuracy, sensitivity, and specificity), which are then averaged to obtain the mean values.

### 2.8. SVR analysis

To verify the ability of the identified brain regions to distinguish between SCZ patients and HCs and to increase the reliability of the results, a machine learning classification analysis was conducted. First, mean ReHo values of brain regions with significant differences were extracted after post-hoc *t*-tests. Then, a linear support vector machine (SVM) with a one-against-one classification strategy was used to classify SCZ patients and HCs, performed by the LIBSVM software (Zuo et al., 2013).^2^

## 3. Results

### 3.1. Demographic and clinical characteristics

A total of 56 patients with SCZ and 51 HCs were enrolled in this study and all of them were included in the final analysis. Among the 56 patients with SCZ, 37 patients completed the 3-month follow-up, with the participants who dropped out mainly citing inconvenience during the COVID-19 pandemic. The demographic and clinical data of the participants are presented in Supplementary Table S1, which shows no significant differences in age, sex, BMI, and years of education between the SCZ and control groups. The psychological and cognitive status of the individuals are also provided in Supplementary Tables S2, S3.

### 3.2. The treatment outcome

The clinical characteristics of 37 patients with SCZ who completed the follow-up are shown in Supplementary Table S4. The results indicate a significant reduction in the scores of the Positive Scale, General Psychopathology Scale, and Total score of the PANSS scale. This demonstrates a significant improvement in symptoms among individuals with SCZ following treatment.

### 3.3. ReHo analysis in pre-treatment patients with SCZ and HCs

**Figure 1:**
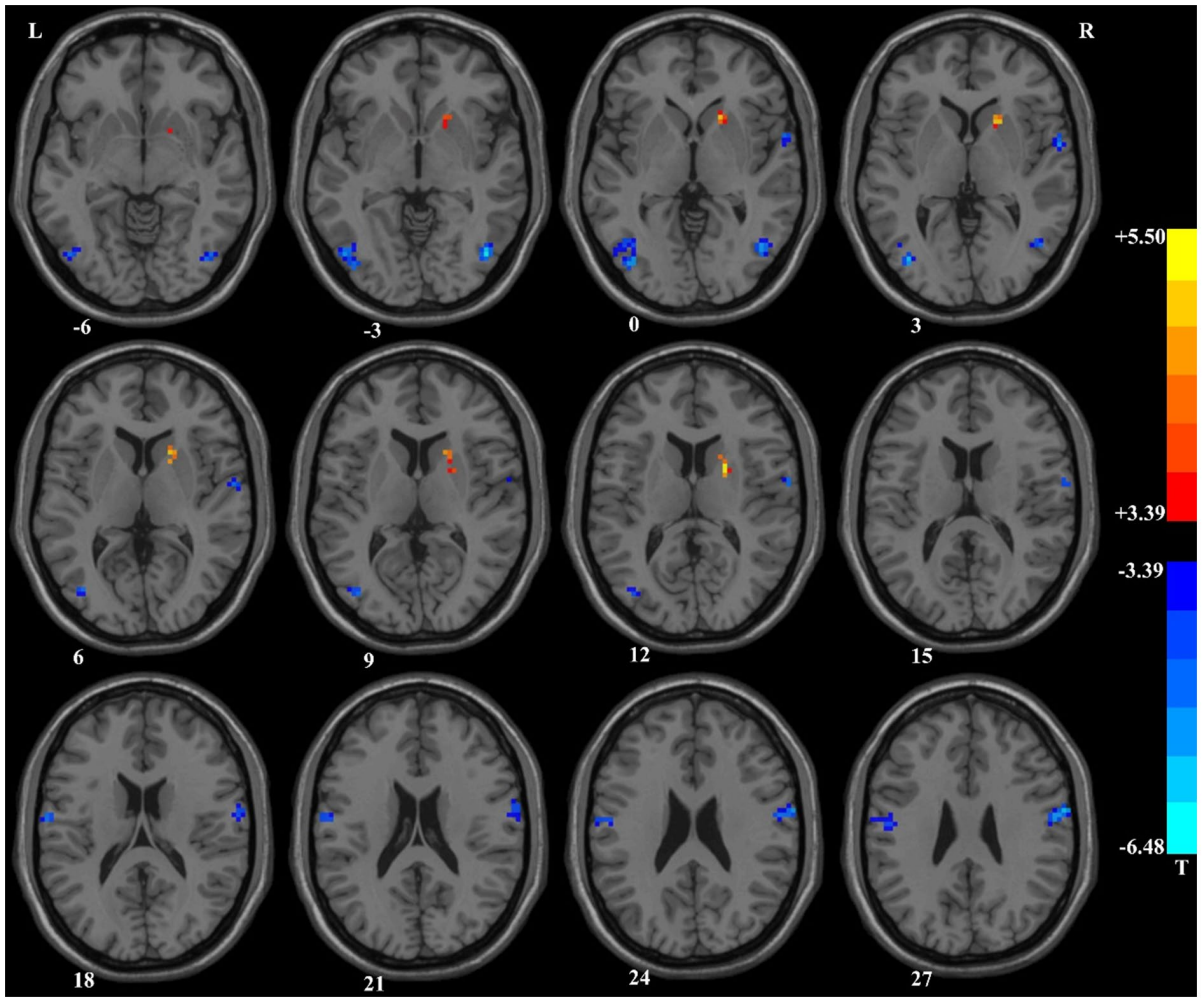
Brain regions with significant differences in the ReHo values at baseline between patients with schizophrenia and healthy controls. ReHo, Regional Homogeneity.

Compared with HCs, patients with SCZ showed lower ReHo values at baseline in several brain regions including the right postcentral/precentral gyrus, left postcentral/inferior parietal gyrus, left middle/inferior occipital gyrus, and right middle temporal/inferior occipital gyrus. In contrast, higher ReHo values were observed in the right putamen at baseline. More detailed information is presented in Table 1 and Figure 1.

**Table 1:** Regions with abnormal ReHo values in patients with schizophrenia at baseline (Figures 1, 2).

Cluster location	Peak (MNI)			Number of voxels	*T* value
	x	y	z		
Patients with schizophrenia at baseline versus controls					
Right postcentral/precentral gyrus	45	−18	33	189	−3.3945
Left postcentral/inferior parietal gyrus	−60	−9	27	179	−3.4070
Left middle/inferior occipital gyrus	−51	−15	33	63	−3.4292
Right middle temporal/inferior occipital gyrus	−45	−24	57	37	−3.4008
Right putamen	21	3	12	34	5.4960
Patients with schizophrenia after 3-month treatment versus at baseline					
Right inferior parietal gyrus	39	−42	45	22	−3.3420

ReHo, Regional Homogeneity; MNI, Montreal Neurological Institute.

### 3.4. Regional homogeneity analysis in pre-treatment and post-treatment patients with SCZ

**Figure 2:**
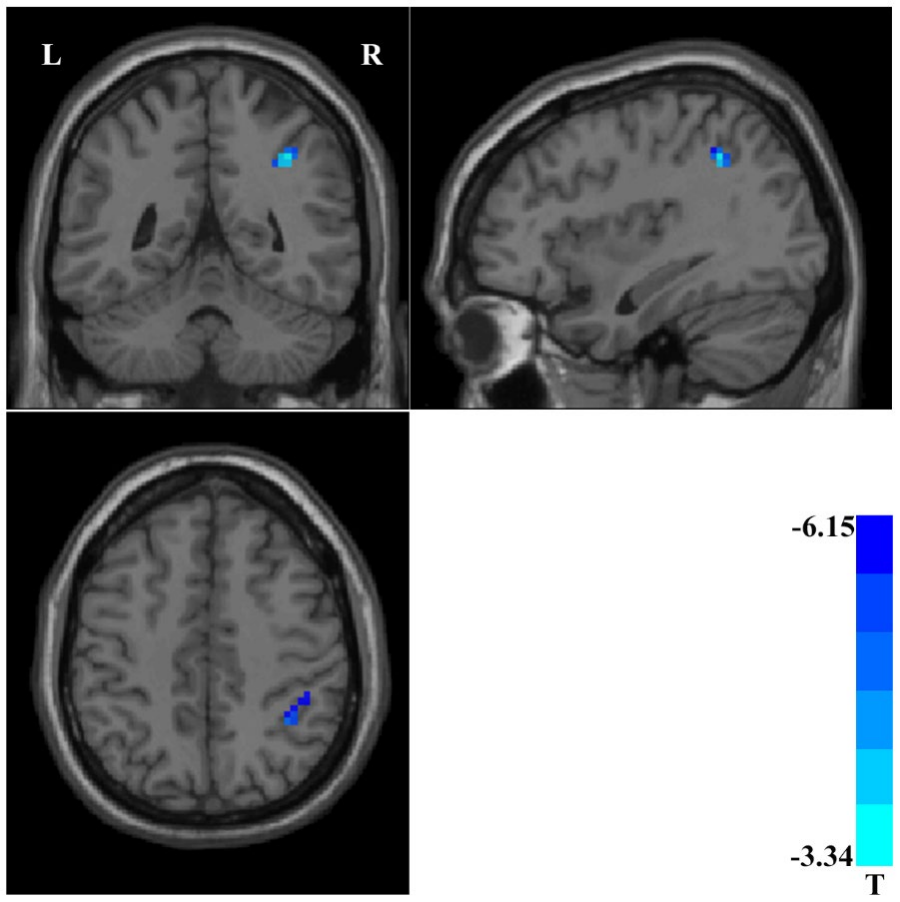
Compared with baseline data, patients with schizophrenia showed significantly decreased ReHo values in the right inferior parietal gyrus after 3-month treatment. ReHo, Regional Homogeneity.

We compared the ReHo values of patients with SCZ who completed follow-up in brain regions with abnormal ReHo values at baseline. Compared with the baseline data, patients with SCZ showed significantly decreased ReHo values after 3-month treatment in the right inferior parietal gyrus (Table 1 and Figure 2).

### 3.5. Support vector machine analysis result

**Figure 3:**
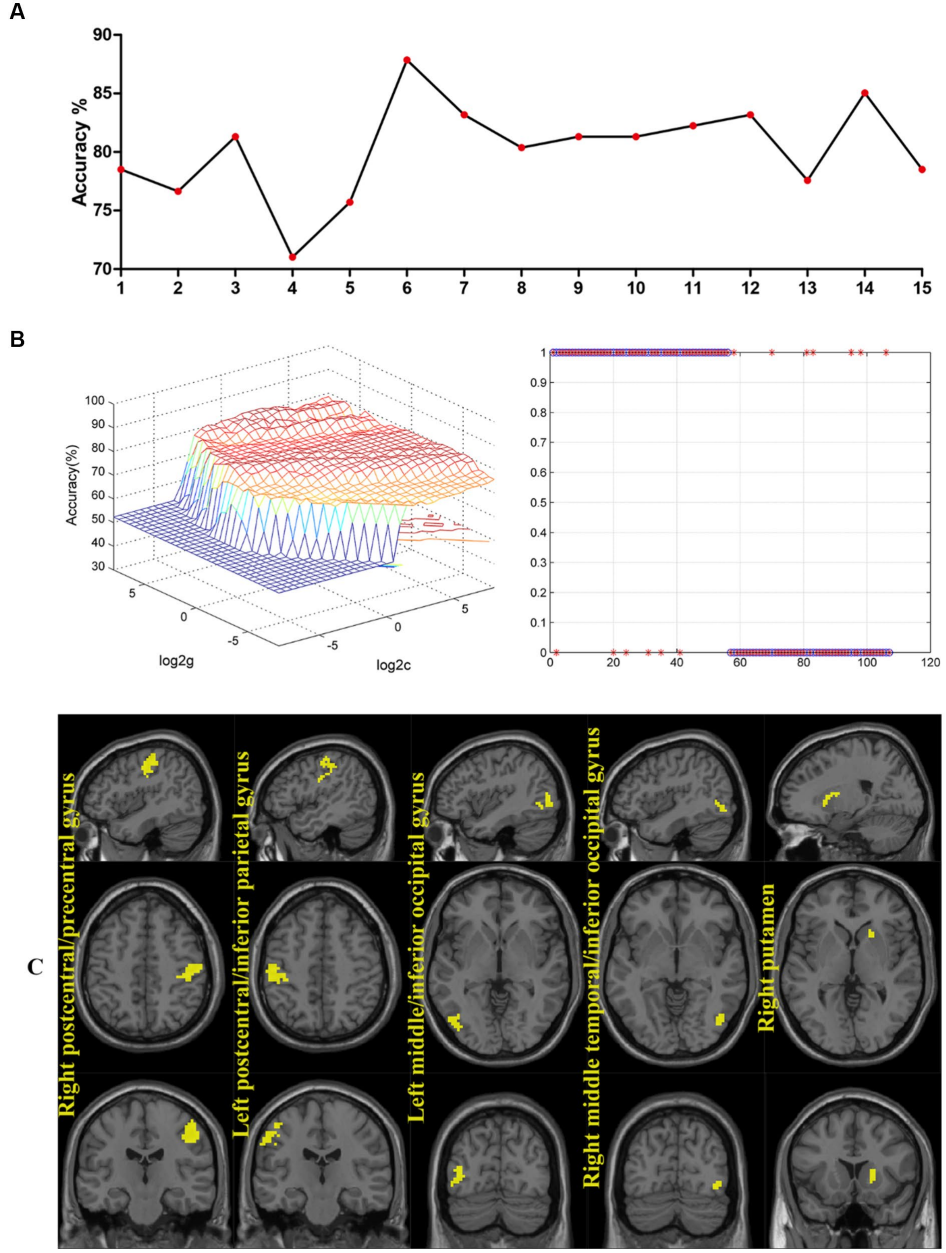
**(A)** The accuracy of classification of SVM applied abnormal ReHo as input. One represents the ReHo values in the right postcentral/precentral gyrus, 2 represents the ReHo values in the left postcentral/inferior parietal gyrus, 3 represents the ReHo values in the left middle/inferior occipital gyrus, 4 represents the ReHo values in the right middle temporal/inferior occipital gyrus, 5 represents the ReHo values in the right putamen, 6 represents the ReHo values in the right postcentral/precentral gyrus and left postcentral/inferior parietal gyrus, 7 represents the ReHo values in the right postcentral/precentral gyrus and left middle/inferior occipital gyrus, 8 represents the ReHo values in the right postcentral/precentral gyrus and right middle temporal/inferior occipital gyrus, 9 represents the ReHo values in the right postcentral/precentral gyrus and right putamen, 10 represents the ReHo values in the left postcentral/inferior parietal gyrus and left middle/inferior occipital gyrus, 11 represents the ReHo values in the left postcentral/inferior parietal gyrus and right middle temporal/inferior occipital gyrus, 12 represents the ReHo values in the left postcentral/inferior parietal gyrus and right putamen, 13 represents the ReHo values in the left middle/inferior occipital gyrus and right middle temporal/inferior occipital gyrus, 14 represents the ReHo values in the left middle/inferior occipital gyrus and right putamen, and 15 represents the ReHo values in the right middle temporal/inferior occipital gyrus and right putamen. **(B)** When the ReHo values in the right postcentral/precentral gyrus and left postcentral/inferior parietal gyrus as the input, the SVM achieved the highest accuracy. Accuracy = 87.85%, Sensitivity = 89.29%, Specificity = 86.27%. SVM, support vector machines; ReHo, Regional Homogeneity. **(C)** Regions with abnormal ReHo values in patients with schizophrenia at baseline.

The accuracy of classification of SVM applied abnormal ReHo as input. When the ReHo values in the right postcentral/precentral gyrus and left postcentral/inferior parietal gyrus as the input, the SVM achieved the highest accuracy. This combination was the optimal combination with an accuracy of 87.85%, a sensitivity of 89.29%, and a specificity of 86.27% (Figure 3).

### 3.6. Support vector regression analysis result

**Figure 4:**
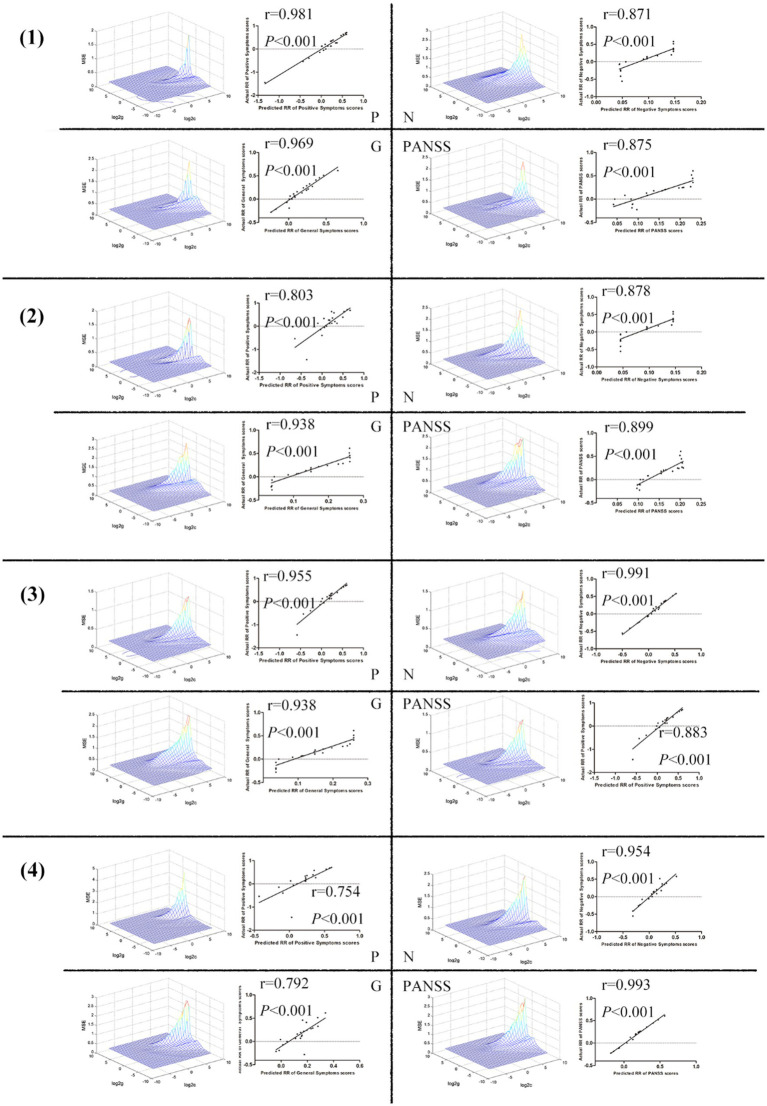
(1) The ReHo values in the right postcentral/precentral gyrus and right middle temporal/inferior occipital gyrus could be used to predict the treatment effects which was reflected by the positive correlation between the predicted and actual reduction rate of the scores of P, N, G, and PANSS. (2) The ReHo values in the left postcentral/inferior parietal gyrus and left middle/inferior occipital gyrus could be used to predict the treatment effects which was reflected by the positive correlation between the predicted and actual reduction rate of the scores of P, N, G, and PANSS. (3) The ReHo values in the left postcentral/inferior parietal gyrus and right putamen could be used to predict the treatment effects which was reflected by the positive FIGURE 4 (Continued)correlation between the predicted and actual reduction rate of the scores of P, N, G, and PANSS. (4) The ReHo values in the right middle temporal/inferior occipital gyrus and right putamen could be used to predict the treatment effects which was reflected by the positive correlation between the predicted and actual reduction rate of the scores of P, N, G, and PANSS. ReHo, Regional Homogeneity; P, Positive Scale; N, Negative Scale; G, General Psychopathology Scale; PANSS, Positive and Negative Syndrome Scale.

The ReHo values in the right postcentral/precentral gyrus and right middle temporal/inferior occipital gyrus, the left postcentral/inferior parietal gyrus and left middle/inferior occipital gyrus, the left postcentral/inferior parietal gyrus and right putamen, and the right middle temporal/inferior occipital gyrus and right putamen could be used to predict the treatment effects which was reflected by the positive correlation between the predicted and actual reduction rate of the scores of P, N, G, and PANSS (Figure 4).

### 3.7. Correlation between regional homogeneity values and clinical symptoms of patients with SCZ

Significant correlations were found between abnormal ReHo values and clinical symptoms in patients with SCZ at baseline. Detailed information is presented in Supplementary Table S5.

## 4. Discussion

In this study, compared to HCs, individuals with SCZ exhibited lower ReHo values in the somatomotor network and higher ReHo values in the right putamen at baseline. After 3 months of treatment, significant clinical improvement was observed in the positive, negative, and general symptoms compared to baseline assessment. Additionally, there was a decrease in ReHo values in the right inferior parietal lobule following treatment compared to baseline measurements. The results of SVR analysis suggested that changes in ReHo values in specific brain regions could be used to predict early treatment response.

### 4.1. Decreased ReHo values in the sensorimotor network in patients with SCZ at baseline

Research has consistently shown that patients with SCZ exhibit lower ReHo values in brain regions related to visual and sensory-motor functions, such as the right postcentral gyrus, left postcentral gyrus/inferior parietal gyrus, left middle/inferior occipital gyrus, and right middle temporal/inferior occipital gyrus, when compared to HCs. Nonetheless, the precise causes of these abnormalities and their connections to the pathophysiology of SCZ remain uncertain and warrant further investigation. Several studies have proposed that abnormalities in neurons, synapses, and neurotransmitters in the brains of individuals with SCZ may play a crucial role in the neuropathology of the disorder (Snyder and Gao, 2013; Grace, 2016; Krajcovic et al., 2019). Neuroimaging methods have offered a potent tool for examining alterations in both functional and structural connectivity within the brains of individuals with SCZ, facilitating a more comprehensive understanding of the disorder (Lahti, 2022). Neurophysiological studies employing transcranial magnetic stimulation have identified abnormalities in cortical excitability within the motor cortex of individuals with SCZ (Fitzgerald et al., 2004; Radhu et al., 2013; Bikson et al., 2016). A research has revealed that patients with SCZ exhibit reduced FC in the motor cortex (Du et al., 2019), as well as in the executive control and auditory networks, when compared to HCs (Joo et al., 2020). Additionally, You et al. (2021) demonstrated a reduction in FC between the visual and executive control networks in patients with SCZ (You et al., 2021). Our study identified decreased ReHo values in the sensorimotor network of SCZ patients compared to HCs, which may contribute to the aberrant daily behaviors observed in these patients.

### 4.2. Increased ReHo values in the right putamen in patients with SCZ at baseline

Anatomically, the putamen is a component of the striatum, a brain region that establishes connections with the internal pallidal segment and substantia nigra pars reticulata (Karnath et al., 2002). Numerous studies have proposed a potential association between the putamen and the pathology of SCZ. These findings suggest that there might be an increase in putamen function during the early stages of the illness (Malaspina et al., 2004; Digney et al., 2005; Kumakura et al., 2007). Furthermore, studies indicate that the enlargement of the putamen in SCZ may serve as a diagnostic characteristic, and a greater putamen volume has been linked to positive treatment outcomes (Buchsbaum et al., 2003; Brickman et al., 2006; Kawasaki et al., 2007). Huang et al. (2010) found that patients with first-episode SCZ exhibited significantly increased amplitude of low-frequency fluctuations (ALFF) in the putamen compared to HCs. We observed an increase in spontaneous brain activity in the right putamen of patients at baseline. Consistent with previous research, there has been a reported a significant increase in FC in the right putamen, regardless of the presence or absence of auditory verbal hallucinations, in individuals with first-episode SCZ (Cui et al., 2016). In our prior research, the patient group exhibited increased functional connectivity in the bilateral putamen at baseline, relative to the control group. Moreover, after 1 week of olanzapine treatment, our study identified a relative decrease in FC in the right putamen compared to the baseline level (Wu et al., 2019). This implies that the increase in ReHo in the right putamen may function as a potential neuroimaging biomarker for the presence of SCZ.

### 4.3. ReHo values in the right inferior parietal gyrus decreased significantly after treatment

The right inferior parietal gyrus is a significant brain region that plays a vital role in numerous cognitive and perceptual processes, encompassing spatial and body awareness, attention, language, and mathematical abilities. Additionally, this region is linked to higher-order cognitive functions such as reasoning, memory, and decision-making. Honea et al.’s (2005) meta-analysis of multiple voxel-based morphometry (VBM) studies identified a reduction in gray matter density and volume in the right inferior parietal gyrus of patients with SCZ. Jung et al.’s (2010) review of MRI studies involving ultra-high risk psychosis patients identified potential abnormalities in the right inferior parietal gyrus. Additionally, Manoach et al.’s (2000) functional MRI (fMRI) study examining working memory tasks in patients with SCZ discovered reduced activation levels in the right inferior parietal gyrus during task performance. In our study, we observed a significant decrease in the ReHo value of the right inferior parietal lobule following 3 months of treatment, suggesting that pharmacological treatment for SCZ might have potential side effects on this brain region.

SVM has emerged as a popular method for classifying mental illnesses. To utilize FC signal as a potential diagnostic index, it is essential to ensure that the sensitivity or specificity of the SVM model is higher than 0.6, which is a commonly used criterion for evaluating the performance of classification models (Guo et al., 2011; Tanskanen et al., 2018; Zhang et al., 2023). Based on the SVM analysis, the ReHo values of the sensorimotor network and the right putamen exhibit a high level of discriminatory power, with sensitivity, accuracy, and specificity exceeding 0.70 when differentiating between SCZ patients and HCs. These findings suggest that SVM has promising potential as a valuable tool for diagnosing and predicting the development of SCZ based on brain imaging data. Additionally, the SVR analysis revealed that the ReHo values of the sensorimotor network and the right putamen could be utilized to predict early treatment response.

This study has several limitations that warrant mention. First, due to the COVID-19 pandemic and individual patient circumstances, the study was conducted at a single center with a relatively small sample size and a high dropout rate, potentially limiting the generalizability of the results to other centers. Second, the follow-up period was only 3 months, which might not be sufficient to observe complete normalization of the decreased ReHo value, as evidenced by the significant decrease in the ReHo value of the right inferior parietal gyrus after treatment. Third, the medication administered to the patients was not restricted to a specific type, and different medications could have varying effects on the results. And we were unable to independently assess the influence of the placebo effect on the results. Fourth, ReHo metric can be affected by amplitude and frequency thresholds. We implemented band-pass filtering within the frequency range of 0.01–0.08 Hz to eliminate noise and unwanted signals while retaining the relevant neural activity of interest. However, we did not apply varying amplitude and frequency thresholds to investigate their potential impact on our findings. Fifth, the resting-state fMRI data exhibit temporal dynamics and are influenced by time. However, in our study, we only conducted baseline scans for HCs and both pre-treatment and post-treatment scans for patients. Consequently, a Two-Way Analysis of Variance could not be performed due to the unequal distribution of scans. As a result, we cannot exclusively attribute the changes in ReHo observed in patients after treatment solely to the treatment effects. Nevertheless, it is worth noting that previous research has established functional connectivity as a relatively stable indicator in resting-state fMRI (Chou et al., 2012). In HCs, the intraindividual fluctuations in functional connectivity between baseline and endpoint are likely to be limited.

## 5. Conclusion

In summary, this study offers groundbreaking insights by comparing changes in ReHo values between HCs and patients with SCZ. The findings indicate that decreased ReHo values in the sensorimotor network and increased ReHo values in the right putamen might be distinctive neurobiological features of SCZ patients, and potential imaging biomarkers for differentiating between HCs and SCZ patients. Furthermore, ReHo values of the sensorimotor network and right putamen could serve as predictors of early treatment response.

## Data availability statement

The original contributions presented in the study are included in the article/Supplementary material, further inquiries can be directed to the corresponding authors.

## Ethics statement

The studies involving humans were approved by the Medical Ethics Committee of Foshan Third People’s Hospital (Foshan Mental Health Center). The studies were conducted in accordance with the local legislation and institutional requirements. The participants provided their written informed consent to participate in this study. Written informed consent was obtained from the individual(s) for the publication of any potentially identifiable images or data included in this article.

## Author contributions

HJ, CZ, and HY: writing—original draft, writing—review and editing, methodology, and software. XL, JL, WL, YO, WW, HG, and WD: validation, investigation, and resources. GX and WG: supervision, project administration, and funding acquisition. All authors contributed to the article and approved the submitted version.

## Funding

This study was supported by grants from the “The 14th Five-Year” Medical High-level Key Medical Specialty Development Project of Foshan (Grant No. FSGSP145069), the project of Foshan Science and Technology Bureau (Grant No. 2020001005608), and National Natural Science Foundation of China (Grant No. 82171508).

## Conflict of interest

The authors declare that the research was conducted in the absence of any commercial or financial relationships that could be construed as a potential conflict of interest.

## Publisher’s note

All claims expressed in this article are solely those of the authors and do not necessarily represent those of their affiliated organizations, or those of the publisher, the editors and the reviewers. Any product that may be evaluated in this article, or claim that may be made by its manufacturer, is not guaranteed or endorsed by the publisher.

## Footnotes

^1^http://www.csie.ntu.edu.tw/~cjlin/libsvm/

^2^Software available at http://www.csie.ntu.edu.tw/~cjlin/libsvm.

## References

[ref1] Acar E. Schenker C. Levin-Schwartz Y. Calhoun V. D. Adali T. (2019). Unraveling diagnostic biomarkers of schizophrenia through structure-revealing fusion of multi-modal neuroimaging data. Front. Neurosci. 13:416. doi: 10.3389/fnins.2019.00416, PMID: 31130835 PMC6509223

[ref2] Bagby R. M. Ryder A. G. Schuller D. R. Marshall M. B. (2004). The Hamilton depression rating scale: has the gold standard become a lead weight? Am. J. Psychiatry 161, 2163–2177. doi: 10.1176/appi.ajp.161.12.2163, PMID: 15569884

[ref3] Ben-Hur A. Ong C. S. Sonnenburg S. Scholkopf B. Ratsch G. (2008). Support vector machines and kernels for computational biology. PLoS Comput. Biol. 4:e1000173. doi: 10.1371/journal.pcbi.1000173, PMID: 18974822 PMC2547983

[ref4] Bikson M. Grossman P. Thomas C. Zannou A. L. Jiang J. Adnan T.. (2016). Safety of transcranial direct current stimulation: evidence based update 2016. Brain Stimul. 9, 641–661. doi: 10.1016/j.brs.2016.06.004, PMID: 27372845 PMC5007190

[ref5] Brickman A. M. Buchsbaum M. S. Ivanov Z. Borod J. C. Foldi N. S. Hahn E.. (2006). Internal capsule size in good-outcome and poor-outcome schizophrenia. J. Neuropsychiatry Clin. Neurosci. 18, 364–376. doi: 10.1176/jnp.2006.18.3.364, PMID: 16963586

[ref6] Buchsbaum M. S. Shihabuddin L. Brickman A. M. Miozzo R. Prikryl R. Shaw R.. (2003). Caudate and putamen volumes in good and poor outcome patients with schizophrenia. Schizophr. Res. 64, 53–62. doi: 10.1016/s0920-9964(02)00526-1, PMID: 14511801

[ref7] Buoli M. Bertino V. Caldiroli A. Dobrea C. Serati M. Ciappolino V.. (2016). Are obstetrical complications really involved in the etiology and course of schizophrenia and mood disorders? Psychiatry Res. 241, 297–301. doi: 10.1016/j.psychres.2016.05.014, PMID: 27232550

[ref8] Chao-Gan Y. Yu-Feng Z. (2010). DPARSF: a MATLAB toolbox for "pipeline" data analysis of resting-state fMRI. Front. Syst. Neurosci. 4:13. doi: 10.3389/fnsys.2010.00013, PMID: 20577591 PMC2889691

[ref9] Chen J. Xu Y. Zhang K. Liu Z. Xu C. Shen Y.. (2013). Comparative study of regional homogeneity in schizophrenia and major depressive disorder. Am. J. Med. Genet. B Neuropsychiatr. Genet. 162, 36–43. doi: 10.1002/ajmg.b.32116 23169775

[ref10] Chou Y. H. Panych L. P. Dickey C. C. Petrella J. R. Chen N. K. (2012). Investigation of long-term reproducibility of intrinsic connectivity network mapping: a resting-state fMRI study. AJNR Am. J. Neuroradiol. 33, 833–838. doi: 10.3174/ajnr.A2894, PMID: 22268094 PMC3584561

[ref11] Cui L. B. Liu K. Li C. Wang L. X. Guo F. Tian P.. (2016). Putamen-related regional and network functional deficits in first-episode schizophrenia with auditory verbal hallucinations. Schizophr. Res. 173, 13–22. doi: 10.1016/j.schres.2016.02.039, PMID: 26995674

[ref12] Dabiri M. Dehghani Firouzabadi F. Yang K. Barker P. B. Lee R. R. Yousem D. M. (2022). Neuroimaging in schizophrenia: a review article. Front. Neurosci. 16:1042814. doi: 10.3389/fnins.2022.1042814, PMID: 36458043 PMC9706110

[ref13] Diaz-Castro L. Hoffman K. Cabello-Rangel H. Arredondo A. Herrera-Estrella M. A. (2021). Family history of psychiatric disorders and clinical factors associated with a schizophrenia diagnosis. Inquiry 58:469580211060797. doi: 10.1177/00469580211060797, PMID: 34845937 PMC8673879

[ref14] Digney A. Keriakous D. Scarr E. Thomas E. Dean B. (2005). Differential changes in apolipoprotein E in schizophrenia and bipolar I disorder. Biol. Psychiatry 57, 711–715. doi: 10.1016/j.biopsych.2004.12.028, PMID: 15820227

[ref15] Du X. Choa F. S. Chiappelli J. Wisner K. M. Wittenberg G. Adhikari B.. (2019). Aberrant middle prefrontal-motor cortex connectivity mediates motor inhibitory biomarker in schizophrenia. Biol. Psychiatry 85, 49–59. doi: 10.1016/j.biopsych.2018.06.007, PMID: 30126607 PMC6289820

[ref16] Fan X. Li H. Lai L. Zhou X. Ye X. Xiao H. (2022). Impact of internet plus health education on urinary stoma caregivers in coping with care burden and stress in the era of COVID-19. Front. Psychol. 13:982634. doi: 10.3389/fpsyg.2022.982634, PMID: 36532976 PMC9748695

[ref17] Fitzgerald P. B. Brown T. L. Marston N. A. Oxley T. De Castella A. Daskalakis Z. J.. (2004). Reduced plastic brain responses in schizophrenia: a transcranial magnetic stimulation study. Schizophr. Res. 71, 17–26. doi: 10.1016/j.schres.2004.01.018 15374568

[ref18] Galinska-Skok B. Waszkiewicz N. (2022). Markers of schizophrenia-a critical narrative update. J. Clin. Med. 11:3964. doi: 10.3390/jcm11143964, PMID: 35887728 PMC9323796

[ref19] Gao B. Wang Y. Liu W. Chen Z. Zhou H. Yang J.. (2015). Spontaneous activity associated with delusions of schizophrenia in the left medial superior frontal gyrus: a resting-state fMRI study. PLoS One 10:e0133766. doi: 10.1371/journal.pone.0133766, PMID: 26204264 PMC4512714

[ref20] Grace A. A. (2016). Dysregulation of the dopamine system in the pathophysiology of schizophrenia and depression. Nat. Rev. Neurosci. 17, 524–532. doi: 10.1038/nrn.2016.57 27256556 PMC5166560

[ref21] Guo W. Liu F. Chen J. Wu R. Li L. Zhang Z.. (2017). Olanzapine modulation of long- and short-range functional connectivity in the resting brain in a sample of patients with schizophrenia. Eur. Neuropsychopharmacol. 27, 48–58. doi: 10.1016/j.euroneuro.2016.11.002 27887859

[ref22] Guo W. B. Liu F. Xue Z. M. Yu Y. Ma C. Q. Tan C. L.. (2011). Abnormal neural activities in first-episode, treatment-naive, short-illness-duration, and treatment-response patients with major depressive disorder: a resting-state fMRI study. J. Affect. Disord. 135, 326–331. doi: 10.1016/j.jad.2011.06.048 21782246

[ref23] Honea R. Crow T. J. Passingham D. Mackay C. E. (2005). Regional deficits in brain volume in schizophrenia: a meta-analysis of voxel-based morphometry studies. Am. J. Psychiatry 162, 2233–2245. doi: 10.1176/appi.ajp.162.12.2233 16330585

[ref24] Huang X. Q. Lui S. Deng W. Chan R. C. Wu Q. Z. Jiang L. J.. (2010). Localization of cerebral functional deficits in treatment-naive, first-episode schizophrenia using resting-state fMRI. NeuroImage 49, 2901–2906. doi: 10.1016/j.neuroimage.2009.11.072, PMID: 19963069

[ref25] Huang Y. Wang W. Hei G. Yang Y. Long Y. Wang X.. (2022). Altered regional homogeneity and cognitive impairments in first-episode schizophrenia: a resting-state fMRI study. Asian J. Psychiatr. 71:103055. doi: 10.1016/j.ajp.2022.103055, PMID: 35303593

[ref26] Hyman S. E. Saha S. Chant D. Welham J. McGrath J. (2005). A systematic review of the prevalence of schizophrenia. PLoS Med. 2:e141. doi: 10.1371/journal.pmed.0020141 15916472 PMC1140952

[ref27] Jauhar S. Johnstone M. McKenna P. J. (2022). Schizophrenia. Lancet 399, 473–486. doi: 10.1016/S0140-6736(21)01730-X 35093231

[ref28] Jiang Y. Xu L. Li X. Tang Y. Wang P. Li C.. (2019). Common increased hippocampal volume but specific changes in functional connectivity in schizophrenia patients in remission and non-remission following electroconvulsive therapy: a preliminary study. Neuroimage Clin. 24:102081. doi: 10.1016/j.nicl.2019.102081, PMID: 31734526 PMC6861644

[ref29] Joo S. W. Yoon W. Jo Y. T. Kim H. Kim Y. Lee J. (2020). Aberrant executive control and auditory networks in recent-onset schizophrenia. Neuropsychiatr. Dis. Treat. 16, 1561–1570. doi: 10.2147/ndt.S254208, PMID: 32606708 PMC7319504

[ref30] Jung W. H. Jang J. H. Byun M. S. An S. K. Kwon J. S. (2010). Structural brain alterations in individuals at ultra-high risk for psychosis: a review of magnetic resonance imaging studies and future directions. J. Korean Med. Sci. 25, 1700–1709. doi: 10.3346/jkms.2010.25.12.1700, PMID: 21165282 PMC2995221

[ref31] Kani A. S. Shinn A. K. Lewandowski K. E. Ongur D. (2017). Converging effects of diverse treatment modalities on frontal cortex in schizophrenia: a review of longitudinal functional magnetic resonance imaging studies. J. Psychiatr. Res. 84, 256–276. doi: 10.1016/j.jpsychires.2016.10.012 27776293 PMC5135290

[ref32] Karnath H. O. Himmelbach M. Rorden C. (2002). The subcortical anatomy of human spatial neglect: putamen, caudate nucleus and pulvinar. Brain 125, 350–360. doi: 10.1093/brain/awf032, PMID: 11844735

[ref33] Kawasaki Y. Suzuki M. Kherif F. Takahashi T. Zhou S. Y. Nakamura K.. (2007). Multivariate voxel-based morphometry successfully differentiates schizophrenia patients from healthy controls. Neuroimage 34, 235–242. doi: 10.1016/j.neuroimage.2006.08.018, PMID: 17045492

[ref34] Kay S. R. Fiszbein A. Opler L. A. (1987). The positive and negative syndrome scale (PANSS) for schizophrenia. Schizophr. Bull. 13, 261–276. doi: 10.1093/schbul/13.2.261 3616518

[ref35] Kemp R. A. Lambert T. J. (1995). Insight in schizophrenia and its relationship to psychopathology. Schizophr. Res. 18, 21–28. doi: 10.1016/0920-9964(95)00018-6 8929757

[ref36] Kozlowska E. Brzezinska-Blaszczyk E. Agier J. Wysokinski A. Zelechowska P. (2021). Alarmins (IL-33, sST2, HMGB1, and S100B) as potential biomarkers for schizophrenia. J. Psychiatr. Res. 138, 380–387. doi: 10.1016/j.jpsychires.2021.04.019, PMID: 33957300

[ref37] Kraguljac N. V. McDonald W. M. Widge A. S. Rodriguez C. I. Tohen M. Nemeroff C. B. (2021). Neuroimaging biomarkers in schizophrenia. Am. J. Psychiatry 178, 509–521. doi: 10.1176/appi.ajp.2020.20030340, PMID: 33397140 PMC8222104

[ref38] Krajcovic B. Fajnerova I. Horacek J. Kelemen E. Kubik S. Svoboda J.. (2019). Neural and neuronal discoordination in schizophrenia: from ensembles through networks to symptoms. Acta Physiol. 226:e13282. doi: 10.1111/apha.13282 31002202

[ref39] Kuloglu M. Bayik Y. Unal A. Gecici O. Ustundag B. (2016). Serum IL-1β, IL-2, IL-6, and IL-8 levels in schizophrenia subtypes. Bull. Clin. Psychopharmacol. 21, 193–200. doi: 10.5455/bcp.20110418011851

[ref40] Kumakura Y. Cumming P. Vernaleken I. Buchholz H. G. Siessmeier T. Heinz A.. (2007). Elevated [18F]fluorodopamine turnover in brain of patients with schizophrenia: an [18F]fluorodopa/positron emission tomography study. J. Neurosci. 27, 8080–8087. doi: 10.1523/JNEUROSCI.0805-07.2007, PMID: 17652599 PMC6672729

[ref41] Lahti A. C. (2022). Discovery of early schizophrenia through neuroimaging. Psychiatry Res. 322:114993. doi: 10.1016/j.psychres.2022.114993, PMID: 36773467 PMC12266044

[ref42] Legge S. E. Santoro M. L. Periyasamy S. Okewole A. Arsalan A. Kowalec K. (2021). Genetic architecture of schizophrenia: a review of major advancements. Psychol. Med. 51, 2168–2177. doi: 10.1017/S0033291720005334, PMID: 33550997

[ref43] Li X. L. Yu Y. Hu Y. Wu H. T. Li X. S. Chen G. Y.. (2022). Fibroblast growth factor 9 as a potential biomarker for schizophrenia. Front. Psych. 13:788677. doi: 10.3389/fpsyt.2022.788677, PMID: 35546939 PMC9082542

[ref44] Ma H. Cheng N. Zhang C. (2022). Schizophrenia and alarmins. Medicina 58:694. doi: 10.3390/medicina58060694, PMID: 35743957 PMC9230958

[ref45] Malaspina D. Harkavy-Friedman J. Corcoran C. Mujica-Parodi L. Printz D. Gorman J. M.. (2004). Resting neural activity distinguishes subgroups of schizophrenia patients. Biol. Psychiatry 56, 931–937. doi: 10.1016/j.biopsych.2004.09.013, PMID: 15601602 PMC2993017

[ref46] Manoach D. S. Gollub R. L. Benson E. S. Searl M. M. Goff D. C. Halpern E.. (2000). Schizophrenic subjects show aberrant fMRI activation of dorsolateral prefrontal cortex and basal ganglia during working memory performance. Biol. Psychiatry 48, 99–109. doi: 10.1016/s0006-3223(00)00227-4, PMID: 10903406

[ref47] Marder S. R. Cannon T. D. (2019). Schizophrenia. N. Engl. J. Med. 381, 1753–1761. doi: 10.1056/NEJMra1808803 31665579

[ref48] Moreno-Kustner B. Martin C. Pastor L. (2018). Prevalence of psychotic disorders and its association with methodological issues. A systematic review and meta-analyses. PLoS One 13:e0195687. doi: 10.1371/journal.pone.0195687, PMID: 29649252 PMC5896987

[ref49] Nieuwenstein M. R. Aleman A. de Haan E. H. (2001). Relationship between symptom dimensions and neurocognitive functioning in schizophrenia: a meta-analysis of WCST and CPT studies. Wisconsin card sorting test. Continuous performance test. J. Psychiatr. Res. 35, 119–125. doi: 10.1016/s0022-3956(01)00014-0, PMID: 11377441

[ref50] Olaithe M. Weinborn M. Lowndes T. Ng A. Hodgson E. Fine L.. (2019). Repeatable battery for the assessment of neuropsychological status (RBANS): normative data for older adults. Arch. Clin. Neuropsychol. 34, 1356–1366. doi: 10.1093/arclin/acy102, PMID: 30608541

[ref51] Onitsuka T. Hirano Y. Nakazawa T. Ichihashi K. Miura K. Inada K.. (2022). Toward recovery in schizophrenia: current concepts, findings, and future research directions. Psychiatry Clin. Neurosci. 76, 282–291. doi: 10.1111/pcn.13342, PMID: 35235256

[ref52] Palacios E. M. Sala-Llonch R. Junque C. Roig T. Tormos J. M. Bargallo N.. (2013). Resting-state functional magnetic resonance imaging activity and connectivity and cognitive outcome in traumatic brain injury. JAMA Neurol. 70, 845–851. doi: 10.1001/jamaneurol.2013.38, PMID: 23689958

[ref53] Perkovic M. N. Erjavec G. N. Strac D. S. Uzun S. Kozumplik O. Pivac N. (2017). Theranostic biomarkers for schizophrenia. Int. J. Mol. Sci. 18:733. doi: 10.3390/ijms18040733 28358316 PMC5412319

[ref54] Radhu N. de Jesus D. R. Ravindran L. N. Zanjani A. Fitzgerald P. B. Daskalakis Z. J. (2013). A meta-analysis of cortical inhibition and excitability using transcranial magnetic stimulation in psychiatric disorders. Clin. Neurophysiol. 124, 1309–1320. doi: 10.1016/j.clinph.2013.01.014 23485366

[ref55] Shan X. Cui X. Liu F. Li H. Huang R. Tang Y.. (2021). Shared and distinct homotopic connectivity changes in melancholic and non-melancholic depression. J. Affect. Disord. 287, 268–275. doi: 10.1016/j.jad.2021.03.038, PMID: 33799047

[ref56] Snyder M. A. Gao W. J. (2013). NMDA hypofunction as a convergence point for progression and symptoms of schizophrenia. Front. Cell. Neurosci. 7:31. doi: 10.3389/fncel.2013.00031, PMID: 23543703 PMC3608949

[ref57] Solmi M. Radua J. Olivola M. Croce E. Soardo L. Salazar de Pablo G.. (2022). Age at onset of mental disorders worldwide: large-scale meta-analysis of 192 epidemiological studies. Mol. Psychiatry 27, 281–295. doi: 10.1038/s41380-021-01161-7 34079068 PMC8960395

[ref58] Song X. W. Dong Z. Y. Long X. Y. Li S. F. Zuo X. N. Zhu C. Z.. (2011). REST: a toolkit for resting-state functional magnetic resonance imaging data processing. PLoS One 6:e25031. doi: 10.1371/journal.pone.0025031, PMID: 21949842 PMC3176805

[ref59] Srivastava A. Dada O. Qian J. Al-Chalabi N. Fatemi A. B. Gerretsen P.. (2021). Epigenetics of schizophrenia. Psychiatry Res. 305:114218. doi: 10.1016/j.psychres.2021.114218 34638051

[ref60] Tanskanen A. Tiihonen J. Taipale H. (2018). Mortality in schizophrenia: 30-year nationwide follow-up study. Acta Psychiatr. Scand. 138, 492–499. doi: 10.1111/acps.12913 29900527

[ref61] Thomas H. V. Dalman C. David A. S. Gentz J. Lewis G. Allebeck P. (2001). Obstetric complications and risk of schizophrenia. Effect of gender, age at diagnosis and maternal history of psychosis. Br. J. Psychiatry 179, 409–414. doi: 10.1192/bjp.179.5.409 11689396

[ref62] Wang Y. Tang W. Fan X. Zhang J. Geng D. Jiang K.. (2017). Resting-state functional connectivity changes within the default mode network and the salience network after antipsychotic treatment in early-phase schizophrenia. Neuropsychiatr. Dis. Treat. 13, 397–406. doi: 10.2147/NDT.S123598 28223812 PMC5308583

[ref63] Wu R. Ou Y. Liu F. Chen J. Li H. Zhao J.. (2019). Reduced brain activity in the right putamen as an early predictor for treatment response in drug-naive, first-episode schizophrenia. Front. Psychiatry 10:741. doi: 10.3389/fpsyt.2019.00741, PMID: 31649567 PMC6791918

[ref64] Xiao B. Wang S. Liu J. Meng T. He Y. Luo X. (2017). Abnormalities of localized connectivity in schizophrenia patients and their unaffected relatives: a meta-analysis of resting-state functional magnetic resonance imaging studies. Neuropsychiatr. Dis. Treat. 13, 467–475. doi: 10.2147/NDT.S126678, PMID: 28243099 PMC5317331

[ref65] Xie Q. Fan F. Fan X. P. Wang X. J. Chen M. J. Zhong B. L.. (2020). COVID-19 patients managed in psychiatric inpatient settings due to first-episode mental disorders in Wuhan, China: clinical characteristics, treatments, outcomes, and our experiences. Transl. Psychiatry 10:337. doi: 10.1038/s41398-020-01022-x, PMID: 33009366 PMC7531059

[ref66] Xu Y. M. Deng F. Zhong B. L. (2022). Facial emotion identification impairments in Chinese persons living with schizophrenia: a meta-analysis. Front. Psych. 13:1097350. doi: 10.3389/fpsyt.2022.1097350 PMC9807786 36606133

[ref67] Yan H. Shan X. Li H. Liu F. Guo W. (2022a). Abnormal spontaneous neural activity as a potential predictor of early treatment response in patients with obsessive-compulsive disorder. J. Affect. Disord. 309, 27–36. doi: 10.1016/j.jad.2022.04.125 35472471

[ref68] Yan H. Shan X. Li H. Liu F. Guo W. (2022b). Abnormal spontaneous neural activity in hippocampal-cortical system of patients with obsessive-compulsive disorder and its potential for diagnosis and prediction of early treatment response. Front. Cell. Neurosci. 16:906534. doi: 10.3389/fncel.2022.906534, PMID: 35910254 PMC9334680

[ref69] Yan W. Zhang R. Zhou M. Lu S. Li W. Xie S.. (2020). Relationships between abnormal neural activities and cognitive impairments in patients with drug-naive first-episode schizophrenia. BMC Psychiatry 20:283. doi: 10.1186/s12888-020-02692-z, PMID: 32503481 PMC7275517

[ref70] You W. F. Luo L. Li F. Gong Q. (2021). Altered brain functional dynamics in auditory and visual networks in schizophrenia. Eur. Psychiatry 64:S159. doi: 10.1192/j.eurpsy.2021.428

[ref71] Yu L. Guo L. Fang X. Yang F. Chen Y. Wang Y.. (2022). Altered brain activity in the bilateral frontal cortices and neural correlation with cognitive impairment in schizophrenia. Brain Imaging Behav. 16, 415–423. doi: 10.1007/s11682-021-00516-6, PMID: 34449034

[ref72] Zang Y. Jiang T. Lu Y. He Y. Tian L. (2004). Regional homogeneity approach to fMRI data analysis. NeuroImage 22, 394–400. doi: 10.1016/j.neuroimage.2003.12.030 15110032

[ref73] Zhang C. Jing H. Yan H. Li X. Liang J. Zhang Q.. (2023). Disrupted interhemispheric coordination of sensory-motor networks and insula in major depressive disorder. Front. Neurosci. 17:1135337. doi: 10.3389/fnins.2023.1135337, PMID: 36960171 PMC10028102

[ref74] Zhu Y. Wang S. Gong X. Edmiston E. K. Zhong S. Li C.. (2021). Associations between hemispheric asymmetry and schizophrenia-related risk genes in people with schizophrenia and people at a genetic high risk of schizophrenia. Br. J. Psychiatry 219, 392–400. doi: 10.1192/bjp.2021.47, PMID: 35048853 PMC8529637

[ref75] Zuo X. N. Xu T. Jiang L. Yang Z. Cao X. Y. He Y.. (2013). Toward reliable characterization of functional homogeneity in the human brain: preprocessing, scan duration, imaging resolution and computational space. Neuroimage 65, 374–386. doi: 10.1016/j.neuroimage.2012.10.017, PMID: 23085497 PMC3609711

