# Supplementary material for: Deviant spontaneous neural activity as a potential early-response predictor for therapeutic interventions in patients with schizophrenia
Source: Front Neurosci. 2023 Aug 31;17:1243168. doi: 10.3389/fnins.2023.1243168 (PMC10505796; doi:10.3389/fnins.2023.1243168)
Supplement: Supplementary file 1 [file Data_Sheet_1.docx]

**Deviant spontaneous neural activity as a potential early-response predictor for therapeutic interventions in patients with schizophrenia**

**Huan Jing ^1^ †, Chunguo Zhang ^1^ †, Haohao Yan^2^ †, Xiaoling Li^1^, Jiaquan Liang^1^, Wenting Liang^1^, Yangpan Ou^2^, Weibin Wu^1^,** **Huagui Guo^1^, Wen Deng^1^, Guojun Xie^1^*, Wenbin Guo^2^***

**Affiliation/address:**

^1^Department of Psychiatry, The Third People's Hospital of Foshan, Foshan, Guangdong 528000, China.

^2^Department of Psychiatry, National Clinical Research Center for Mental Disorders, and National Center for Mental Disorders, The Second Xiangya Hospital of Central South University, Changsha 410011, Hunan, China.

†: Huan Jing, Chunguo Zhang and Haohao Yan contributed equally to this work.

***Correspondence:**

Wenbin Guo

Department of Psychiatry, National Clinical Research Center for Mental Disorders, and National Center for Mental Disorders, The Second Xiangya Hospital of Central South University, Changsha 410011, Hunan, China.

Email: [guowenbin76@csu.edu.cn](mailto:guowenbin76@csu.edu.cn)

Guojun Xie

Department of Psychiatry, The Third People's Hospital of Foshan, Foshan, Guangdong 528000, China.

Email: xiegjfs@126.com

TableS1. Demography and clinical characteristics

| Variables | Patients (56) | Controls (51) | *p*-value |
| --- | --- | --- | --- |
| Age (years) | 41.76±10.13 | 41.00±10.78 | 0.708^a^ |
| Sex (male/female) | 24/32 | 19/32 | 0.349^b^ |
| Years of education (years) | 10.33±3.11 | 11.61±3.64 | 0.054^a^ |
| BMI（kg/m^2^） | 24.30±4.51 | 24.16±3.40 | 0.859^a^ |
| TSH3UL（mIU/L） | 2.27±1.79 | 2.40±1.07 | 0.648^a^ |
| FT3（pmol/L） | 4.38±0.80 | 4.76±0.57 | 0.005^a^ |
| FT4（pmol/L） | 15.41±4.79 | 15.00±3.05 | 0.608^a^ |
| TG | 1.67±1.84 | 2.02±2.80 | 0.461^a^ |
| CHOL | 4.34±0.78 | 4.89±0.85 | 0.001^a^ |
| HDL | 1.07±0.31 | 1.15±0.30 | 0.169^a^ |
| LDL | 2.46±0.51 | 2.71±0.66 | 0.034^a^ |
| FBG | 5.33±0.81 | 5.75±0.95 | 0.017^a^ |
| Cortisol | 373.23±119.41 | 300.67±107.28 | 0.002^a^ |
| Uric acid | 355.83±102.11 | 354.42±94.51 | 0.942^a^ |
| HR（times/minute） | 74.18±11.67 | 67.29±10.22 | <0.001^a^ |
| QRS width(ms） | 92.48±10.88 | 96.63±10.97 | 0.053^a^ |
| PR interval（ms） | 144.73±19.95 | 157.18±17.85 | 0.001^a^ |
| QTc（ms） | 378.70±34.36 | 397.73±23.83 | 0.001^a^ |

BMI= Body Mass Index; TSH3UL=Thyroid Stimulating Hormone; FT3=Free Triiodothyronine; FT4=Free Thyroxine; TG=triglyceride; CHOL=Cholesterol; HDL=High Density Lipoprotein; LDL=Low Density Lipoprotein; FBG=Fasting Blood Glucose; HR=Heart Rate.

^a^ The *p*-values were obtained by two sample *t*-tests.

^b^ The *p*-value for sex distribution was obtained by a chi-square test.

TableS2. Comparison of patients and healthy controls in psychological status

| Variables | Patients (n = 56) | Controls (n = 51) | *p*-value |
| --- | --- | --- | --- |
| **PANSS** |  |  |  |
| P | 16.04±6.92 | 7.00±0.00 | <0.001^a^ |
| N | 22.64±5.61 | 7.04±0.28 | <0.001^a^ |
| G | 33.00±7.44 | 16.16±0.88 | <0.001^a^ |
| Total score | 71.67±14.96 | 30.20±1.15 | <0.001^a^ |
| HAMD | 6.04±5.27 | 2.98±4.01 | 0.001^a^ |
| HAMA | 4.56±3.66 | 2.67±3.09 | 0.005^a^ |
| **SCSQ** |  |  |  |
| active coping | 19.14±8.70 | 23.04±6.33 | 0.009^a^ |
| negative coping | 9.45±4.75 | 7.78±4.38 | 0.065^a^ |
| total score | 28.59±12.09 | 30.82±8.07 | 0.265^a^ |
| **SDSS** | 8.87±3.03 | 0.02±0.14 | <0.001^a^ |

PANSS=Positive and Negative Syndrome Scale; P=Positive Scale; N=Negative Scale; G=General Psychopathology Scale; SCSQ=Simplified Coping Style Questionnaire; SDSS=Social Disability Screening Schedule;

^a^ The *p*-values were obtained by two sample *t*-tests.

TableS3. Comparison of patients and healthy controls in cognitive status

| Tests | Patients (n = 56) | Controls (n = 51) | *p*-value |
| --- | --- | --- | --- |
| **WCST** |  |  |  |
| CC | 1.70±1.89 | 4.76±1.61 | <0.001^a^ |
| RA | 47.56±1.67 | 45.38±3.34 | <0.001^a^ |
| RC | 16.52±11.37 | 32.56±7.43 | <0.001^a^ |
| RE | 31.04±12.22 | 12.82±9.33 | <0.001^a^ |
| RP | 15.50±14.01 | 3.68±4.69 | <0.001^a^ |
| RPE | 1.17±1.83 | 1.02±1.48 | 0.655^a^ |
| **RBANS** |  |  |  |
| 1A | 17.11±6.63 | 25.50±6.41 | <0.001^a^ |
| 1B | 5.56±4.68 | 11.42±4.90 | <0.001^a^ |
| 2 | 14.11±5.13 | 17.00±2.88 | 0.001^a^ |
| 3 | 11.60±4.42 | 17.80±3.87 | <0.001^a^ |
| 4A | 10.55±2.52 | 13.26±2.66 | <0.001^a^ |
| 4B | 27.96±12.49 | 46.96±13.13 | <0.001^a^ |
| 5A | 3.00±2.42 | 5.84±3.05 | <0.001^a^ |
| 5B | 17.04±3.73 | 20.12±4.43 | <0.001 ^a^ |
| 5C | 2.76±3.03 | 6.14±3.16 | <0.001 ^a^ |
| 5D | 7.89±5.34 | 13.32±4.29 | <0.001 ^a^ |
| **SCWT** |  |  |  |
| At | 75.28±37.13 | 53.57±10.92 | <0.001 ^a^ |
| Bt | 130.57±59.46 | 81.25±19.06 | <0.001 ^a^ |
| Ct | 234.19±87.08 | 145.21±40.73 | <0.001 ^a^ |
| (C-B)/A | 1.49±0.79 | 1.20±0.56 | 0.034 ^a^ |
| C-2B+100 | 66.84±66.72 | 83.63±34.20 | 0.109 ^a^ |
| **Error reaction** |  |  |  |
| Ae(missay) | 1.43±2.78 | 0.18±0.56 | 0.002 ^a^ |
| Ae(correction) | 1.75±1.87 | 0.96±1.11 | 0.010 ^a^ |
| Ae(block) | 0.11±0.375 | 0.22±0.51 | 0.229 ^a^ |
| Ae(total) | 3.30±4.14 | 1.36±1.32 | 0.002 ^a^ |
| Be(missay) | 3.25±6.51 | 0.98±1.52 | 0.017 ^a^ |
| Be(correction) | 3.38±2.96 | 2.12±1.85 | 0.011 ^a^ |
| Be(block) | 0.58±1.42 | 0.96±1.83 | 0.246 ^a^ |
| Be(total) | 7.21±7.50 | 4.06±3.78 | 0.008 ^a^ |
| Ce(missay) | 8.70±15.13 | 2.52±3.22 | 0.005 ^a^ |
| Ce(correction) | 5.48±3.61 | 3.62±3.19 | 0.007 ^a^ |
| Ce(block) | 1.28±2.63 | 2.54±3.50 | 0.041 ^a^ |
| Ce(total) | 15.45±15.29 | 8.68±7.58 | 0.005 ^a^ |
| (C-B)/A | 2.51±3.91 | 2.06±2.93 | 0.521 ^a^ |
| C-2B+100 | 101.04±9.61 | 100.80±5.77 | 0.879 ^a^ |

WCST = Wisconsin card sorting test; CC = Categories Completed; RA = Responses Answer; RC = Correct Responses; RE = Errors Responses; RP = Perseverative Responses; RPE = Perseverative Responses Errors; RBANS = Repeatable Battery for the Assessment of Neuropsychological Status; NEF = number of eye fixation; RSS = responsive search score; D=Discriminant analysis; SCWT=Stroop color word test.

^a^ The *p*-values were obtained by two sample *t*-tests.

Table S4. Characteristics of patients who finished the follow-up.

| Variables | Pre-treatment (Mean ± SD, n=37) | Post-treatment (Mean ± SD, n=37) | *p* |
| --- | --- | --- | --- |
| Age (years) | 42.43±9.50 |  |  |
| Sex (male/female) | 16/21 |  |  |
| Years of education (years) | 10.32±2.93 |  |  |
| PANSS |  |  |  |
| P | 15.05±5.01^b^ | 11.59±4.91^b^ | 0.034^a^ |
| N | 23.18±4.25^b^ | 20.50±5.50^b^ | 0.079^a^ |
| G | 33.95±6.91^b^ | 27.55±4.72^b^ | 0.004^a^ |
| Total score | 72.18±12.96^b^ | 59.64±11.90^b^ | 0.004^a^ |
| HAMD | 5.62±5.20^b^ | 6.24±5.32^b^ | 0.742^a^ |
| HAMA | 4.33±3.38^b^ | 4.00±4.10^b^ | 0.798^a^ |
| ITAQ | 3.55±3.17^b^ | 4.50±4.21^b^ | 0.335^a^ |

SD = Standard Deviation; PANSS = Positive and Negative Syndrome Scale; P = Positive Scale; N = Negative Scale; G = General Psychopathology Scale; HAMD = Hamilton Depression Rating Scale; HAMA = Hamilton Anxiety Rating Scale; ITAQ=Insight and Treatment Attitudes Questionnaire.

^a^ The *p*-values were obtained by paired *t*-tests.

^b^ The data were obtained from 22 patients with schizophrenia.

Table S5: Correlation between ReHo values and the clinical data.

| ReHo values in the brain region | Clinical data | r | *p* |
| --- | --- | --- | --- |
| Right postcentral/precentral gyrus | General Psychopathology Scale | 0.274 | 0.043 |
|  | PANSS total | 0.291 | 0.031 |
|  | RBANS 2 | 0.278 | 0.040 |
|  | RBANS 3 | 0.328 | 0.015 |
|  | Bt | 0.352 | 0.010 |
|  | C-2B+100 | -0.277 | 0.045 |
|  | Ae(correction) | 0.274 | 0.047 |
|  | RPE | 0.287 | 0.035 |
|  | NEF | 0.281 | 0.046 |
| Left postcentral/inferior parietal gyrus | Ae(correction) | 0.349 | 0.010 |
|  | C-2B+100_2 | 0.348 | 0.011 |
| Left middle/inferior occipital gyrus | C-2B+100_2 | 0.283 | 0.040 |
| Right middle temporal/inferior occipital gyrus | P200 | -0.325 | 0.017 |
| Right putamen | Negative Scale | -0.273 | 0.043 |
|  | RBANS_3 | 0.328 | 0.015 |

PANSS = Positive and Negative Syndrome Scale; RBANS = Repeatable Battery for the Assessment of Neuropsychological Status; RPE = Perseverative Responses Errors; NEF = number of eye fixation.
